# Safety of home-based exercise for people with intermittent claudication: A systematic review

**DOI:** 10.1177/1358863X211060388

**Published:** 2021-12-20

**Authors:** Alexander Waddell, Sally Seed, David R Broom, Gordon McGregor, Stefan T Birkett, Amy E Harwood

**Affiliations:** 1 Centre for Sport, Exercise and Life Sciences, Institute of Health and Wellbeing, Coventry University, Coventry, UK; 2 School of Sport and Health Sciences, University of Central Lancashire, Preston, UK; 3 Department of Cardiopulmonary Rehabilitation, Centre for Exercise & Health, University Hospitals Coventry & Warwickshire NHS Trust, Coventry, UK; 4 Warwick Clinical Trials Unit, Warwick Medical School, University of Warwick, Coventry, UK

**Keywords:** claudication, complication rate, exercise therapy, home-based exercise, peripheral artery disease (PAD), safety

## Abstract

Intermittent claudication (IC) is a classic symptom of peripheral artery disease, with first line treatment being supervised exercise therapy (SET). Despite this, SET is frequently underutilised, and adherence is often poor. An alternative option are home-based exercise programmes (HBEP). Although HBEPs are well tolerated, to the authors’ knowledge, no research has assessed their safety. The aim of this review was to assess the safety of HBEPs in people living with IC. We performed an electronic search of the MEDLINE, CINAHL, and Cochrane Library databases. The main parameter of interest was complication rate, calculated as the number of related adverse events per patient-hours. Subanalysis was undertaken to determine differences in safety for studies that did and did not include pre-exercise cardiac screening, and for studies with exercise at low, moderate, and high levels of claudication pain. Our search strategy identified 8693 results, of which 27 studies were included for full review. Studies included 1642 participants completing 147,810 patient-hours of home-based exercise. Four related adverse events were reported, three of which were cardiac in origin, giving an all-cause complication rate of one event per 36,953 patient-hours. Three of these events occurred following exercise to high levels of claudication pain, and one occurred with pain-free exercise. One event occured in a study without cardiac screening. Based on the low number of related adverse events, HBEPs appear to be a safe method of exercise prescription for people with IC. Our results strengthen the rationale for providing alternative exercise options for this population. **PROSPERO Registration No.: CRD42021254581**

## Background

Peripheral artery disease (PAD) affects over 236 million people worldwide.^1^ Intermittent claudication (IC) is a classic symptom of PAD, defined as calf pain or discomfort induced by exertion which is relieved by rest.^2^ IC is associated with an increased risk of mortality,^3^ and reductions in quality of life,^4^ functional ability,^5^ and physical activity behaviour.^6^

National and international guidelines recommend supervised exercise therapy (SET) as first line treatment for IC.^7,8^ Structured SET has shown improvements in peak oxygen consumption, pain-free and maximal walking distances, and quality of life.^9,10^ Despite strong evidence demonstrating the benefits of SET, its availability, adherence, and uptake remain low,^11–13^ especially in the US, when compared to antiplatelet therapy, statins, and smoking cessation.^14^ Commonly cited barriers include time constraints, travel difficulties, and other personal commitments.^15,16^

To improve the access and availability of exercise programmes for people with IC, studies have investigated the role of home-based exercise programmes (HBEP). Although HBEPs are well tolerated and have shown improvements in walking performance,^17^ to our knowledge, no research has assessed their safety. Supervised exercise therapy has already been deemed safe for those with IC, with a low complication rate and with no need for prior cardiac screening.^18^ Unlike SET in a clinical setting or part of an exercise referral scheme, there is no direct supervision from a health or fitness professional when undertaking a HBEP, which may increase the risk of exercise-related complications and compromise safety. Some HBEPs involve remote exercise monitoring, such as with activity watches or telephone contact, which improves the effectiveness of HBEPs,^17^ but does not necessarily monitor participant safety.

The aim of this review, therefore, was to assess the safety of HBEPs in people living with IC by calculating a complication rate.

## Methods

This review was conducted in line with the Preferred Reporting Items for Systematic Reviews and Meta-Analyses (PRISMA) guidelines.^19^ The systematic review was registered with PROSPERO (Registration No.: CRD42021254581).

### Eligibility

We included studies that investigated the effects of HBEPs in people diagnosed with symptomatic IC secondary to PAD (Fontaine II / Rutherford 1–3). Studies that included other PAD subgroups, such as asymptomatic individuals, were only included where specific data were obtained on the IC subgroup. We included studies examining HBEPs alone, in comparison to other forms of exercise or to nonexercise controls. Both randomised and nonrandomised studies were included. Studies were limited to those in humans and written in English. There was no restriction on publication date.

### Search

We performed an electronic search of the MEDLINE, CINAHL, and Cochrane Library databases up to June 2021. Reference lists of retrieved studies were also examined along with ClinicalTrials.gov registrations for trials in progress. The search included MeSH terms and keywords relating to the topic of the review, which can be found in the online supplementary material. Examples include: ‘Peripheral Arterial Disease’, ‘intermittent claudication’, ‘PAD’, ‘home-based exercise’, ‘home-based training’, and ‘walking programme’.

### Data extraction and management

Search results were first imported into the reference management software Endnote (version X9.3.3, Clarivate Analytics) for duplicate removal, before being transferred to Covidence (version v2698, Veritas Health Innovations) for screening. Titles and abstracts of imported studies were reviewed independently by two researchers (AW and SS), with conflicts being resolved by a third (AH). The full texts of included studies were subsequently reviewed and data were collected on the study population, number of participants, frequency, duration, and content of exercise sessions, number of adverse events, and drop out/loss to follow up. If data were unclear regarding adverse events or reasons for drop out, the authors were contacted for clarification. If no clarification was received, these studies were excluded, a decision based on the methods of Gommans et al.^18^

## Outcomes

The main outcome of interest was complication rate, calculated using Microsoft Excel as the number of adverse events deemed directly related to exercise participation, per patient-hours. The number of patient-hours was calculated as the number of participants multiplied by the number of exercise sessions, corrected for the average session duration.^18^ Studies that did and did not include pre-exercise cardiac screening were separated to assess any differences in exercise-related event occurrence. This was also performed for studies with exercise prescribed at low, moderate and high levels of claudication pain.

## Risk of bias

Depending on the research design, bias was assessed using either the ‘Revised Cochrane Risk-of-Bias Tool for Randomised Trials’^20^ or the ‘Risk Of Bias in Non Randomised Studies – of Interventions’ (ROBINS-I).^21^ Risk of bias was assessed independently by two reviewers (AW and SS), with disagreements settled with discussion or by a third reviewer (AH). Bias was classified as either low, some concerns, or high.

## Results

### Study selection

A search of the literature identified a total of 8693 studies, of which 27 were included in the full review following screening (online supplementary material). Twelve of the included studies were randomised controlled trials,^22–33^ and the remaining 15 were nonrandomised.^34–48^ The included studies comprised a total of 2351 participants, 1642 of whom received a home-based intervention, representing a total of 147,810 patient-hours.

### Participants

The mean age of participants was 67 ± 2 years, ranging from 64 to 72 years, and, on average, 67% were male. All studies included participants with symptomatic IC. One study also included atypical leg symptoms, such as ischemic leg pain in the buttocks or thigh.^26^ In the included studies, common reasons for excluding participants were a walking limitation other than PAD, having had a revascularisation procedure in the last 3 months, foot ulcers, critical limb ischemia, active cancer treatment, an inability to walk unaided, and ischemic pain at rest.

### Study characteristics

Ten of the included studies were performed in North America, with the remaining studies being conducted across Western Europe (Table 1). Study duration ranged from 4 to 52 weeks, with a 12-week programme being the most common (11/27 studies). Exercise session duration was between 30 and 60 minutes and were completed over 3–7 days per week. Exercise programmes in all the included studies consisted of walking, with one utilising music to guide participants,^34^ one using walking poles,^33^ and one using resistance band exercises in addition to walking.^35^ The description of exercise location was varied, including at home, an unsupervised setting, in the community, a self-chosen environment, an outdoor track, or was not specifically stated. Exercise intensity was generally prescribed via claudication pain. We separated programmes based on the degree of claudication pain exercise was prescribed at. Six of the studies described the claudication pain as near maximal, severe or intense, and were classed as ‘high pain’.^23–26,39,46^ There were nine programmes with exercise that was pain-free, near the pain threshold, or at maximal asymptomatic speed, which were classed as ‘low pain’.^26,28,32,33,42–45,47^ Nine studies included exercise to moderate pain levels.^27,29,30,35–38,40,41^ Two studies did not provide a clear description of the degree of claudication pain^22,34^ and two did not report this information.^31,48^

**Table 1.** Characteristics of studies included in review.

Author (year)	Participants (*n*)	Age (y)	Male (%)	Study arms	Exercise program				Adverse events	Main findings
					Claudication pain	Programme duration (weeks)	Session frequency (days/week)	Session duration (mins)		
Gardner (2011)^23^	119	65	48	HBW, SET, usual care	High	12	3	42	1 myocardial infarction	HBEP and SET groups significantly improved COT (*p* < 0.001) and PWT (*p* < 0.01)
Gardner (2014)^22^	180	66	53	HBW, SET, light resistance	Low-moderate	12	3	38	None	HBEP and SET groups significantly improved COT, PWT, 6MWD, daily average cadence
Savage (2001)^24^	21	66	71	HBW, SET	High	24	3	27.5	None	HBEP and SET groups significantly improved ACD (*p* < 0.01)
McDermott (2013)^25^	194	70	50	HBW, education control	High	24	5	50	1 dyspnoea on exercise	HBEP group significantly improved 6MWD (*p* < 0.001) compared to control group
McDermott (2021)^26^	305	69	52	High-intensity HBW, low-intensity HBW	High and low	52	5	50	1 cardiac arrhythmia, 1 chest pain during exercise	Low-intensity group did not improve 6MWD (*p* = 0.34), high-intensity group significantly improved (*p* < 0.001)
Sandercock (2007)^27^	43	65	74	HBW, SET, usual care	Moderate	12	3	30	None	SET group significantly improved MWT (*p* < 0.001)
Van Schaardenburgh (2017)^28^	29	68	55	HBW, calf raises	Low	8	3	30	None	Calf raise group significantly improved PFWD (*p* = 0.04) and MWD (*p* = 0.47)
Mays (2015)^29^	25	65	80	HBW, walking advice	Moderate	12	3	42.5	None	HBEP group did not improve PWT (*p* = 0.052) compared to control
Regensteiner (1997)^30^	20	64	NR	HBW, hospital-based walking	Moderate	12	3	42.5	None	Hospital-based group significantly improved COT, PWT, V̇O_2max_ (*p* < 0.05)
Collins (2011)^31^	145	66	69	HBW, attention control	NR	24	3	50	None	No significant change in MWD (*p* = 0.60)
Lamberti (2016)^32^	27	69	78	Metronome instructed HBW, revascularisation	Low	16	6	20	None	Both groups significantly improved QoL, ICD, ACD, and PFWD
Spafford (2014)^33^	52	65	67	HBW, Nordic pole walking	Low	12	3	30	None	Nordic pole group significantly improved PFWD (*p* < 0.001) and MWD (*p* = 0.001)
Bronas (2019)^34^	11	65	67	Music guided HBW	Low-moderate	12	3	45	None	6MWD significantly improved at 6 weeks (*p* < 0.001) and 12 weeks (*p* = 0.001)
Cornelis (2021)^35^	20	64	75	HBW and resistance bands	Moderate	4	5	30	None	High participant satisfaction with programme
Degischer (2002)^36^	59	68	64	HBW, SET, SET with clopidogrel	Moderate	12	7	60	None	All groups significantly improved ICD (*p* < 0.05); both SET groups improved ACD (*p* < 0.05)
Dopheide (2015)^37^	40	NR	NR	HBW	Moderate	52	4	45	None	Significantly improved MWD and reduced ROS production (*p* < 0.01), and increased anti-inflammatory profile (*p* < 0.0001)
Dopheide (2017)^38^	60	68	70	HBW, SET, usual care	Moderate	28	4	45	None	SET group significantly increased femoral artery lumen diameter (*p* = 0.03)
Fakhry (2011)^39^	217	68	62	HBW, supervised exercise	High	24	7	30	None	HBEP group significantly improved PFWD (*p* = 0.03) and MWD (*p* < 0.01)
Gyldenløve (2019)^40^	28	68	67	HBW	Moderate	12	7	30	None	Significant improvements in PFWD (*p* = 0.02) and MWD (*p* = 0.03); no change in SO_2_ at 12 weeks
Imfeld (2006)^41^	55	69	62	HBW, SET, SET with clopidogrel	Moderate	12	7	60	None	All groups significantly improved physical function SF-36 scores
Lamberti (2021)^42^	83	72	78	Metronome-instructed HBW	Low	36	6	16	None	Significant improvement in PFWD (*p* < 0.001) but not 6MWD
Malagoni (2011)^43^	250	70	76	Metronome-instructed HBW	Low	52	6	20	None	Significant improvements in SF-36 domains and walking performance (*p* < 0.0001)
Manfredini (2004)^44^	29	66	66	Metronome-instructed HBW	Low	17	7	26	None	Significant improvements in PTS, S_max_ (*p* < 0.02) and ABPI (*p* = 0.01)
Manfredini (2008)^45^	126	68	81	Metronome instructed HBW	Low	24	6	20	None	Significant improvement in pain symptoms, ICD and ACD (*p* < 0.0001)
Mouser (2009)^46^	120	67	66	HBW	High	24	4	30	None	Significant improvement in ICD (*p* = 0.001) and ACD (*p* = 0.006)
Prévost (2015)^47^	46	60	87	HBW	Low	52	3	35	None	Significant improvement in SF-36 scores (*p* < 0.05), ICD and ACD (*p* < 0.01)
Roberts (2008)^48^	47	67	70	HBW	NR	12	7	60	None	Significant improvement in MWD, VascuQol, peripheral haemodynamics and lactate levels (*p* < 0.001)

ABPI, ankle-brachial pressure index; ACD, absolute claudication distance; COT, claudication onset time; HBEP, home-based exercise programmes; HBW, home-based walking; ICD, initial claudication distance; MWD, maximum walking distance; 6MWD, 6-minute walk distance; NR, not reported; PFWD, pain-free walking distance; PTS, pain threshold speed; PWT, peak walking time; QoL, quality of life; ROS, reactive oxygen species; SET, supervised exercise therapy; SF-36, 36-item short-form survey; S_max_, maximum speed; SO_2_, oxygen saturation; V̇O_2max_; maximal oxygen uptake.

### Adverse events

Potentially related adverse events were clearly reported in 14 of the included studies.^22,23,25,26,28,31–33,36,39,41–43,46^ The remaining 13 studies reported no events.^24,27,29,30,34,35,37,38,40,44,45,47,48^ Four related adverse events occurred in total (three cardiac and one noncardiac in origin) resulting in a total all-cause event rate of one event per 36,953 patient-hours (Table 2). The total cardiac and noncardiac event rates were one per 49,270 and one per 147,810 patient-hours, respectively (Table 2). Three of the events occurred in a programme prescribing exercise to high levels of claudication pain, and one event in a programme at low pain levels. Eight studies employed pretraining cardiac screening consisting of cardiopulmonary exercise testing (CPET) with electrocardiography (ECG) and/or expired gas analysis.^24,27–30,44^ There were no related adverse events reported in studies employing pretraining cardiac screening.

**Table 2.** Comparison of complication rates when undertaking a supervised or home-based exercise programme.

Complication rates	Supervised^a^	Home-based
All-cause	1:10,340	1:36,953
Cardiac	1:13,788	1:49,270
Noncardiac	1:41,363	1:147,810

a Supervised exercise complication rates taken from Gommans et al.^18^

### Risk of bias

Risk of bias assessments are reported in the online supplementary material. Some concerns were raised in five studies due to inadequate information regarding the randomisation process^24,30^ or the allocation sequence concealment.^28,31,33^ Two studies were deemed at high risk of bias due to missing data, with an imbalance in reasons for withdrawal in one^36^ and a large amount of imputed data in another.^32^ Five studies raised some concerns over the possibility of measurement of the outcome being influenced by knowledge of group allocation, as they required judgements from participants aware of their intervention.^32,35,41,43,47^ Three studies were deemed at high risk of bias due to selective reporting, with two missing questionnaire data^22,23^ and one not reporting all time points for treadmill data.^41^ One study was deemed at high risk of bias due to unclear group definition,^44^ and another raised some concerns as groups were created retrospectively.^42^ Two studies raised some concerns over deviations from the intervention, due to an imbalance in medication between groups creating a potential co-intervention^38^ and the intervention not being delivered successfully in the majority of participants.^46^

## Discussion

The aim of this review was to assess the safety of HBEPs in people living with IC by determining the number of exercise-related adverse events. Previous research has indicated that SET is safe in this population;^18^ however, to our knowledge, this is the first study to replicate these methods in HBEPs. In total, there were four related adverse events during 147,000 patient-hours of exercise, resulting in an all-cause event rate of 1 per 36,953 patient-hours.

The all-cause event rate associated with HBEPs was considerably lower than SET, which has previously been reported as 1 per 10,340 patient-hours.^18^ Furthermore, the cardiac event rate following HBEPs was lower than in SET (Table 2). There appears to be fewer adverse events after completing a HBEP, despite a potentially greater duration and frequency of training. However, this is likely a result of the exercise being completed at a self-selected pace (despite prescribed intensity), and thus participants may be less likely to experience an exercise-induced event. Despite this, in some cases, the safety of HBEPs may have been overestimated. The number of patient-hours was calculated based on the number and duration of training sessions prescribed. However, not all participants complied with this prescription, which may mean the number of events underrepresents the true risk of full participation. Additionally, owing to the lack of supervision, it is likely that events may have not always been reported by participants.

Gommans et al. determined there were no differences in the number of events when including or not including pre-exercise CPET and ECG;^18^ thus, the authors of the review deemed it not to be clinically relevant. Although the American Heart Association recommends exercise testing to identify any exercise-induced coronary ischaemia,^49^ existing best practice guidelines do not currently advocate CPETs in those with PAD.^50^ In our review, three of the four events occurred in studies employing pre-exercise cardiac screening using ECG. More extensive screening may be more relevant prior to commencing HBEPs than SET, as participants are exercising alone without regular heart rate or blood pressure monitoring. In this case, more care may be needed to account for occult coronary artery disease, which may be exposed following the exercise programme as exercise tolerance improves. However, this was not evident from our analysis, as a lack of screening beforehand did not result in any more adverse events.

Three events were seen following exercise to high claudication pain^23,25,26^ and the remaining event occurred following pain-free exercise.^26^ This may highlight concerns as the expanding literature of HBEPs covers exercise prescribed at a range of claudication pain levels. For example, the current guidelines recommend walking to moderate-maximal claudication pain,^7,8^ and there is increasing interest in programmes prescribing pain-free walking.^26,39,51^ It is worth noting that by monitoring exercise intensity via claudication pain, higher overall exercise workloads are likely not reached as individuals are symptom-limited before reaching higher equivalents of heart rate or oxygen uptake during which risk may be greater. However, previous research has shown that exercising at heart rates equivalent to claudication pain onset provides a tolerable stimulus that exceeds the anaerobic threshold, thus promoting cardiovascular and metabolic adaptions.^52^ This is demonstrated by the majority of included studies having resulted in significant improvements to walking performance. Asymptomatic individuals may be able to reach higher workloads without being limited by claudication pain, and so may be at a greater risk of cardiovascular events than those with classic IC symptoms. This is important when considering the safety of exercise for PAD in the wider population, as classic IC (calf cramp and pain during exertion) is not experienced by all people living with PAD.^53^

Interestingly, despite the exercise programmes in the included studies being described as home-based, the location of the exercise varied. Locations mentioned included: in the home, the community, in a chosen environment, or on an outdoor track, with the majority not giving a clear description. It can be argued that exercise that does not take place inside or within the vicinity of the home (including driveways and gardens) is not home-based. Related adverse events occurring during exercise that do not fall in line with this definition would therefore not represent the safety of a HBEP. Differences in external factors such as the quality of the walking surface, access to areas to sit down, obstacles that exacerbate leg pain, and weather will not be consistent and may potentially influence the risk to the participant’s safety.^54^

### Study limitations

This review is not without limitations. The description of exercise programmes was unclear at times making it difficult to ascertain if the programme was really home-based. The accuracy of calculating the total number of patient-hours was impeded by lack of clarity, including some programmes increasing exercise session duration over the course of the programme but not providing specifics. Also, frequency of training was prescribed and reported as a range rather than as a specific number of days per week. Although potentially related adverse events were reported in 14/27 studies, the relatedness was decided by our interpretation on a number of occasions. Therefore, some events we deemed unrelated may in fact have been due to exercise, leading to the potential for some misclassification. Not all authors of studies with potentially related events responded to our emails, meaning certain HBEPs which may have altered the results had to be excluded. Additionally, strict participant exclusion criteria likely reduce the ability to generalise the results. As this clinical population tend to be highly co-morbid, an overestimation of the safety of exercise programmes may be potentially harmful. As improvements to exercise tolerance occur, patients may begin to experience previously unseen symptoms of coronary artery disease, presenting further concerns over safety. However, owing to such a low event rate, it is unlikely that the increase in adverse events, when not excluding more at-risk participants, will outweigh the benefits to walking performance, mortality or quality of life seen with exercise programmes. Despite the limitations of our review, we are confident in the findings owing to rigorously following standardised operating procedures and reaching consensus at times of conflict and uncertainty.

## Conclusion

The present review adds to existing evidence regarding the safety of exercise in people living with IC. The complication rates associated with HBEPs are lower than those with SET, which were already reported to be extremely low. Given that patients are exercising at home unsupervised, pre-exercise cardiac screening via CPETs may be more advisable in HBEPs than SET; however, this difference was not discernible from our analysis due to the lack of programmes in this review that included a CPET and a low adverse event rate. Prescribing exercise based on claudication pain level likely means workloads where participants are at greater risk are not reached. Future research should, therefore, assess whether exercise in asymptomatic patients who may be able to tolerate these greater workloads, is as safe as in those with IC.

## Supplemental Material

sj-pdf-1-vmj-10.1177_1358863X211060388 – Supplemental material for Safety of home-based exercise for people with intermittent claudication: A systematic review Click here for additional data file. Supplemental material, sj-pdf-1-vmj-10.1177_1358863X211060388 for Safety of home-based exercise for people with intermittent claudication: A systematic review by Alexander Waddell, Sally Seed, David R Broom, Gordon McGregor, Stefan T Birkett and Amy E Harwood in Vascular Medicine
