# Supplementary material for: Safety of home-based exercise for people with intermittent claudication: A systematic review
Source: Vasc Med. 2021 Dec 20;27(2):186–92. doi: 10.1177/1358863X211060388 (PMC8996308; doi:10.1177/1358863X211060388)
Supplement: sj-pdf-1-vmj-10.1177_1358863X211060388 – Supplemental material for Safety of home-based exercise for people with intermittent claudication: A systematic review [file sj-pdf-1-vmj-10.1177_1358863X211060388.pdf]

Supplementary Online Content

Item 1. Review search terms
Item 2. PRISMA flow diagram
Item 3. Risk of Bias for Randomised Controlled Trials
Item 4. Risk of Bias for Non-Randomised Controlled Trials

This supplementary material has been provided by the authors as additional information.

Item 1. Search criteria

“Peripheral Arterial Disease”[Mesh] OR “peripheral arterial disease” OR “peripheral artery disease” OR “peripheral arterial obstructive disease” OR “peripheral occlusive disease” OR “peripheral vascular disease” OR “intermittent claudication” OR “claudication” OR “lower extremity arterial disease” OR “PAD”

AND

“Exercise”[Mesh] OR “home-based” OR “home-training” OR “home-based exercise” OR “home-based training” OR “home-based exercise programme” OR “home-based exercise program” OR “home-based training programme” OR “home-based training program” OR “home-based walking” OR “walking” OR “walking programme” OR “walking program” OR “non-supervised exercise” OR “unsupervised exercise” OR “community-based” OR “community-based exercise” OR “community-based walking” OR “physical activity” OR “physical activity programme” OR “physical activity program” OR “Wearable Electronic Devices”[Mesh] OR “telemonitoring” OR “mhealth” OR “ehealth” OR “remote monitoring”

Item 2. Preferred Reporting Items for Systematic reviews and Meta Analyses (PRISMA) flow diagram for study selection of the current review

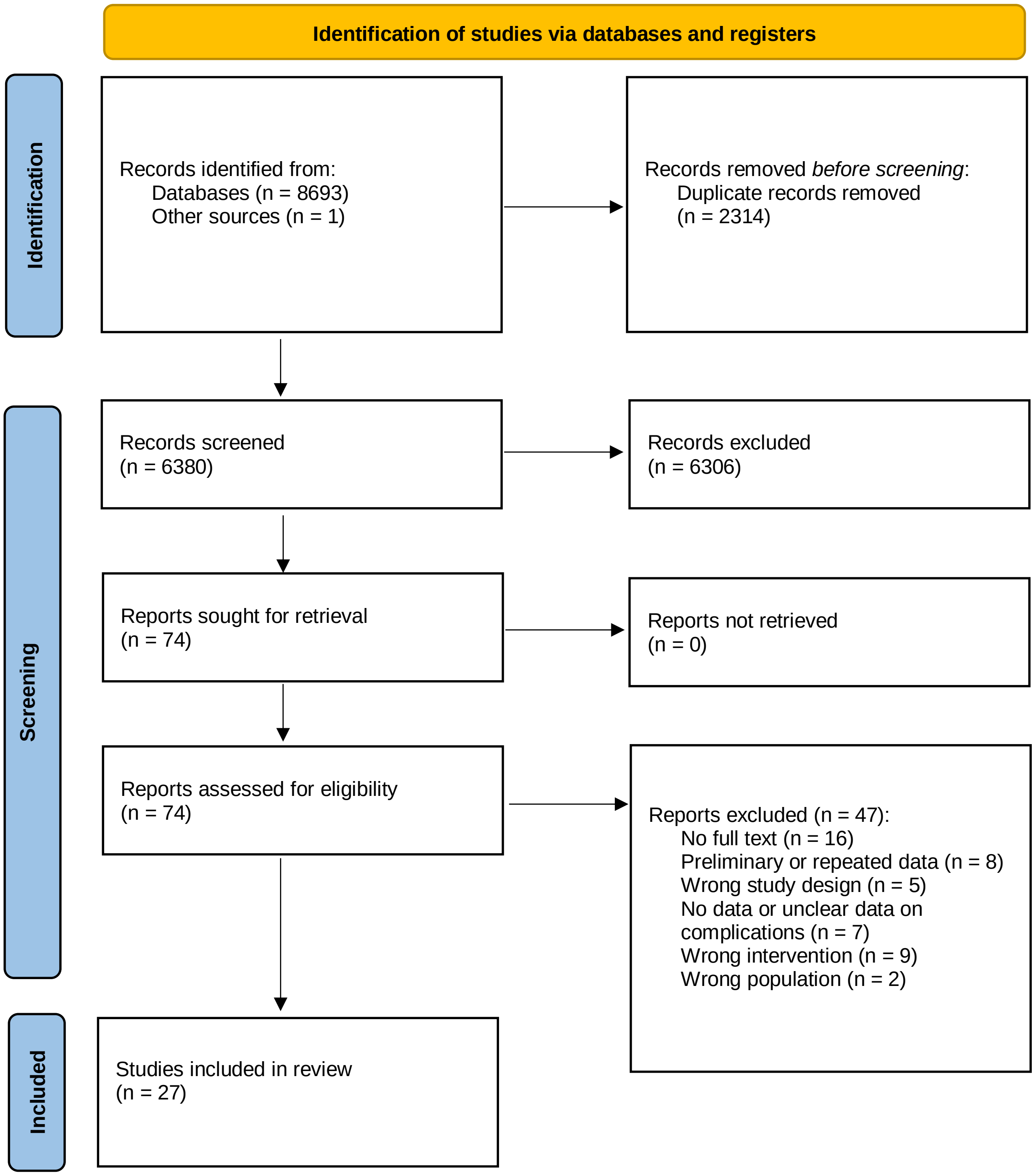

Item 3. Risk of Bias summary showing authors agreed judgement for each domain for included randomised controlled trials. + = low risk, ? = some concerns, - = high risk

	Randomization process	Deviations from intended interventions	Missing outcome data	Measurement of the outcome	Selection of the reported result	Overall
Gardner, 2011[23]	+	+	+	+	–	–
Gardner, 2014[22]	+	+	+	+	–	–
Savage, 2001[24]	?	+	+	+	+	?
McDermott, 2013[25]	+	+	+	+	+	+
McDermott, 2021[26]	+	+	+	+	+	+
Sandercock, 2007[27]	+	+	+	+	+	+
VanSchaardenburgh, 2017[28]	?	+	+	+	+	?
Mays, 2015[29]	+	+	+	+	+	+
Regensteiner, 1997[30]	?	+	+	+	+	?
Collins, 2011[31]	?	+	+	+	+	?
Lamberti, 2016[32]	+	+	–	?	+	–
Spafford, 2014[33]	?	+	+	+	+	?

+ Low risk

? Some concerns

– High risk

Item 4. Risk of Bias summary showing authors agreed judgement for each domain for included non-randomised trials. + = low risk, ? = some concerns, - = high risk

	Bias due to confounding	Bias due to selection of participants	Bias in the classification of interventions	Bias due to deviations from intended interventions	Bias due to missing data	Bias in the measurement of outcomes	Bias in selection of the reported results	Overall
Bronas, 2019[34]	+	+	+	+	+	+	+	+
Cornelis, 2021[35]	+	+	+	+	+	?	+	?
Degischer, 2002[36]	+	+	+	+	–	+	+	–
Dopheide, 2015[37]	+	+	+	+	+	+	+	+
Dopheide, 2017[38]	+	+	+	?	+	+	+	?
Fakhry, 2011[39]	+	+	+	+	+	+	+	+
Gyldenlove, 2019[40]	+	+	+	+	+	+	+	+
Imfeld, 2006[41]	+	+	+	+	+	?	–	–
Lamberti, 2021[42]	+	+	?	+	+	+	+	?
Malagoni, 2011[43]	+	+	+	+	+	?	+	?
Manfredini, 2004[44]	+	+	–	+	+	+	+	–
Manfedini, 2008[45]	+	+	+	+	+	+	+	+
Mouser, 2009[46]	+	+	+	?	+	+	+	?
Prevost, 2015[47]	+	+	+	+	+	?	+	?
Roberts, 2008[48]	+	+	+	+	+	+	+	+

+ Low risk

? Some concerns

– High risk
